# Current Status of Dermatophytosis: A Hospital-Based Study in Northern Odisha, India

**DOI:** 10.7759/cureus.48664

**Published:** 2023-11-11

**Authors:** Sambit Ranjan Dalei, Debabrata Nayak, Pradip Kumar Bhue, Nikhil Ranjan Das, Binodini Behera

**Affiliations:** 1 Dermatology, Fakir Mohan Medical College, Balasore, IND; 2 Dermatology, Pandit Raghunath Murmu Medical College, Baripada, IND; 3 Community Medicine, Government Medical College & Hospital, Sundargarh, IND; 4 Dermatology, Venereology, and Leprology, Saheed Laxman Naik Medical College, Koraput, IND

**Keywords:** northen india, tertiary hospital, retrospective study, dermatophytosis, current status

## Abstract

Introduction

Dermatophytosis is a superficial mycosis that affects the keratinized structures of skin, hair, and nails. In recent years, there has been a marked rise in superficial fungal infections in several parts of our country. Most of the cases are presented with chronic, recurrent, and resistant infections. There are no comprehensive data on the status of cases in our area. Currently, we have noticed a gradual decline in the number of cases in our day-to-day outpatient department compared to previous years. Therefore, the current retrospective study was conducted to assess the actual status and clinico-demographic trends of dermatophytosis among patients who visited the tertiary care hospital.

Methods

The current study is a retrospective study of 78,028 patients of dermatophytosis who reported to the tertiary care hospital. Data like demographic details, clinical examination findings, and laboratory investigation reports of patients were extracted from available records year-wise from 2018 to 2022.

Results

There was an initial rise of dermatophytosis until 2019 and, thereafter, a downward trend. Males (60.04%) outnumbered females (39.94%) with a proportion of 1.5:1. Most common age group belongs to 21-30 years (28.64%), of which a majority (53.07%) of patients had a disease duration of more than three months. The majority of patients (51.1%) belong to rural backgrounds, but there was urban predominance following the coronavirus disease (COVID-19) pandemic. The most common organism isolated from culture was *Trichophyton mentagrophytes* (61.98%) in the initial phases (2018 and 2019) and *T. rubrum* (31.67%) in the later phases (2020-2022). In the current study, a family history of dermatophytosis was present in 27% of cases. Tinea corporis was the most common (34.34%) clinical variant with atopic diathesis as a major co-morbidity (9.94%).

Conclusion

The findings of this study showed an initial increasing trend until 2019 and, thereafter, a downward trend. Therefore, similar types of studies may be carried out in different parts of the country to assess the actual status and, hence, a better and more efficient management of the disease.

## Introduction

Dermatophytes are superficial mycoses that affect the keratinized structures, i.e., hair, nails, and epidermis [1]. The culprit fungi are *Microsporum*, *Trichophyton*, and *Epidermophyton*. Morphologically, these fungi are septate hyphae molds that reproduce asexually in warm environments [2].

In recent years, there has been a dramatic rise in cases of dermatophytosis in different parts of India. Most of the patients are presenting with chronic, recurrent, and treatment-resistant dermatophytosis. Hot and humid climatic conditions of India, coupled with overcrowding, poor socioeconomic conditions, and inadequate hygiene, increase the chances of dermatophytic infection [3]. Other contributing factors that are fueling its rise are host susceptibility to infection, i.e., diabetes, immuno-suppression, atopic dermatitis, agent virulence, and resistance to anti-fungal drugs, and nevertheless an irrational combination of topical steroid cream, particularly clobetasol mixed with other antibacterial and antifungal medications. Due to these contributing factors, most of the patients are getting distressed not only socially and emotionally but also financially [4].

In the recent past, the emergence of the coronavirus disease (COVID- 19) pandemic in the year 2020 has caused unprecedented devastation in people’s lives. The magnitude of its impact is not fully realized until today. Fortunately, the health measures taken during the pandemic, such as using face masks, social distancing, and frequent hand washing, may have impacted the transmission of various communicable diseases, including dermatophytosis [5].

Hence, this study was conducted to assess the burden and clinical picture of dermatophytosis in a tertiary hospital, as there are currently no such data available.

## Materials and methods

The present study was a retrospective study undertaken by the Department of Dermatology and Venereology at the Fakir Mohan Medical College and Hospital, Balasore, India. The study was conducted after receiving approval from the institutional ethical committee (60/IEC dated 15/02/23). A total of 78,028 patients were included in the study using the following criteria.

Inclusion criteria

Patients diagnosed with dermatophytosis between January 1, 2018, and December 31, 2022, with complete records were included in the study.

Exclusion criteria

Patients with incomplete records were excluded from the study.

Study tool

A data abstraction form was prepared as per the study objectives to collect the data from the records of the dermatology department. It includes four sections, i.e., socio-demographic information, patients’ complete history, clinico-dermatological findings, and laboratory results of patients. In socio-demographic information, data regarding the age, sex, residence, and occupation of patients were included. Patients’ complete history included both personal and family history of dermatophytosis, disease durations, and associated co-morbidities, i.e., diabetes mellitus, hypertension, thyroid disease, atopic diathesis, anemia, COPD, CKD, and liver diseases. Clinico-dermatological findings included different clinical variants of dermatophytosis, i.e., tinea cruris, tinea corporis, tinea cruris et corporis, tinea faciei, tinea unguium, tinea capitis, erythroderma, tinea pedis, tinea manuum, and tinea incognito. Laboratory investigations included complete blood count, blood sugar, liver function test, renal function test, thyroid profile, and microbiological study scrapings and/or clippings of skin, hair, or nails.

Data collection method

Using the data abstraction form, the data of 78,028 dermatophytosis patients were extracted from different registers of the dermatology department from January 1, 2018, to December 31, 2022. The study variables, such as socio-demographic information, complete patient history, and clinical and dermatological findings, were extracted for all the patients of dermatophytosis. In addition to this, in chronic and recurrent cases, laboratory investigations, such as complete blood count, blood sugar, liver function test, renal function test, and thyroid profile, were done. In cases where clinical diagnosis was not possible, microbiological study of specimens, i.e., skin, nail, and hair scrapings and/or clippings, was done. The specimens were examined using a 10% potassium hydroxide (KOH) solution to see the presence or absence of fungal elements. In treatment-resistant cases for organism identification, specimens were inoculated on Sabouraud dextrose agar with 0.05% chloramphenicol and 0.5% cycloheximide at 25°C for a duration of up to four weeks.

Data analysis

After data collection, the data obtained were cleaned, compiled, and tabulated year-wise from 2018 to 2022. Data were analyzed with the help of IBM SPSS Statistics, version 21.0 (IBM Corp., Armonk, NY). All the descriptive data were presented with frequency and percentage.

## Results

From 2018 to 2022, 78,028 patients were enlisted for the study. In 2018, 21,769 patients of dermatophytosis were enrolled, whereas the number of patients increased to 25,344 in 2019. In 2020, the number of patients showed a sharp decline up to 14,192. However, 9437 and 7286 patients with dermatophytosis were reported in 2021 and 2022, respectively.

**Figure 1:**
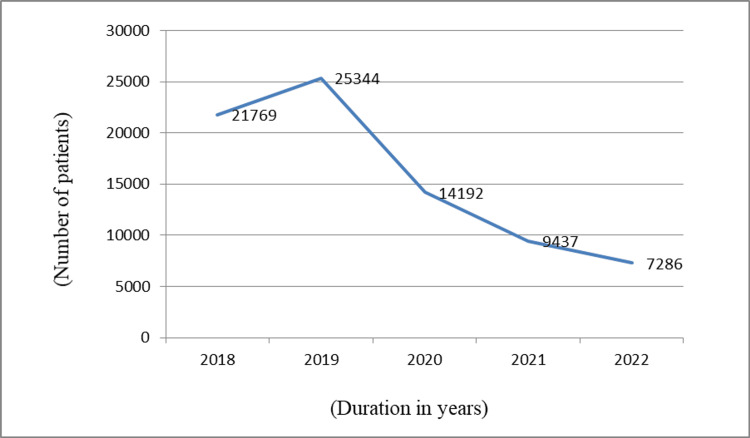
Trend of dermatophytosis patients from 2018 to 2022

There was an increase in the number of cases of dermatophytosis in 2019 compared to 2018, but from 2020 and subsequently in 2021 and 2022, a downward curve was seen, as shown in Figure 1.

Among 78,028 patients, males were 46,846 (60.04%) and females were 31,182 (39.96%). Males outnumbered females with a proportion of 1.5:1. The profile of patients whose data have been taken over the years with gender distribution is shown in Table 1. 

**Table 1:** Profile of patients over the years with gender distribution

Study period	2018	2019	2020	2021	2022
No. of patients	21,769	25,344	14,192	9437	7286
Male	11756	14409	8516	5756	4209
Female	9013	9735	5676	3681	3077

**Figure 2:**
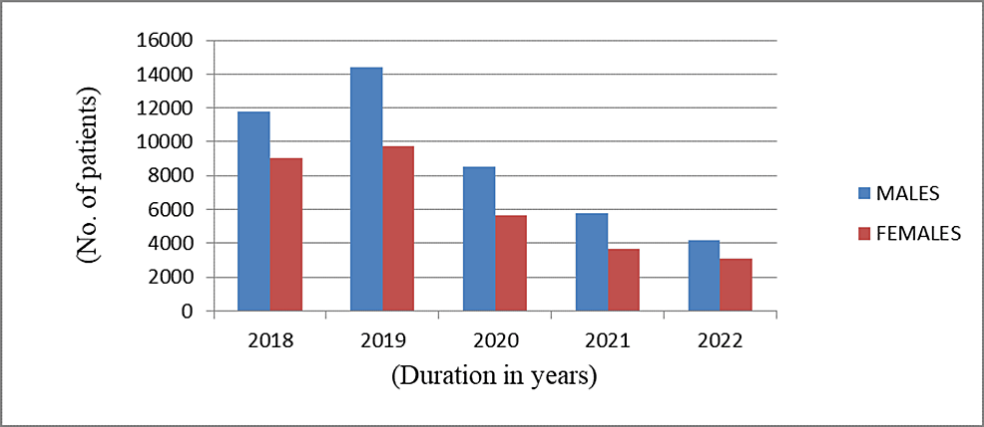
Gender distribution of patients over the years

The gender distribution of all 78,028 patients attending the facility is shown in Figure 2, showing male predominance. 

Most of the cases of dermatophytosis were in the age group of 21-30 years (28.64%), followed by the age group of 31-40 years (24.25%), as depicted in Table 2.

**Table 2:** Age distribution of dermatophytosis patients

Age group	2018	2019	2020	2021	2022
0-10	719	945	458	216	189
11-20	1894	2256	1097	754	678
21-30	6345	6743	4324	3173	2466
31-40	5538	5987	2981	2455	2259
41-50	5211	5893	3177	1652	1177
>50	2062	3520	2155	1187	585

The majority of patients (53.07%) had a disease duration of more than three months, as represented in Table 3. 

**Table 3:** Disease duration among patients

Disease duration (in months)	2018	2019	2020	2021	2022
<1	3608	3905	3634	2747	2206
1-3	5129	6098	3715	3128	2445
>3	13,032	15,341	6843	3562	2635

**Figure 3:**
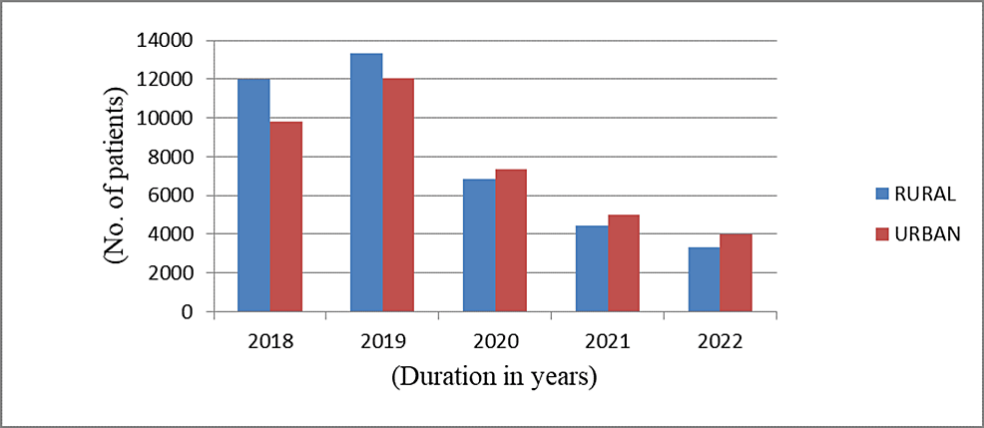
Urban/rural population distribution among patients

The rural population was the major contributor of patients in 2018 and 2019. However, the urban population overcame the rural counterpart from 2020 onward, as shown in Figure 3. Still, the majority of the patients (51.1%) belonged to the rural population.

Laborers and farmers (30%) were the common occupational groups affected by dermatophytes in 2018 and 2019, but from 2020 onward, unemployed people (students and homemakers) were predominantly (37.37%) affected, as shown in Table 4. But, overall farmers and laborers (29.72%) were the most common diseased occupation. 

**Table 4:** Occupation of patients of dermatophytosis

Occupation	2018	2019	2020	2021	2022
Business	3268	4211	2113	1402	1054
Independent profession	6723	7023	3289	1872	1282
Farmer	3789	4011	1997	1289	1054
Laborer	3478	3567	1654	1335	1021
Unemployed	4511	6523	5139	3539	2875

Tinea corporis (34.34%) was the most common variant observed, followed by the mixed variety (22.34%) of both tinea cruris and tinea corporis, as shown in Table 5.

**Table 5:** Clinical variant among patients of dermatophytosis

Clinical variant	2018	2019	2020	2021	2022
Tinea cruris	3145	4765	2769	2339	1326
Tinea corporis	7773	8234	4887	3109	2798
Tinea cruris et corporis	5635	6401	2464	1534	1398
Tinea faciei	1534	1730	1143	632	569
Tinea unguium	1007	1309	590	488	245
Tinea capitis	351	287	233	207	105
Erythroderma	316	376	211	108	96
Tinea pedis	410	485	239	134	81
Tinea manuum	359	412	229	141	76
Tinea incognito	1239	1345	1427	745	592

Over the years, 27% of patients had at least one family member affected by dermatophytosis. Atopic diathesis (9.94%) was the major co-morbidity, followed by diabetes, hypertension, and thyroid diseases, as shown in Table 6.

**Table 6:** Co-morbidities among dermatophytosis patients

Co-morbidities	2018	2019	2020	2021	2022
Diabetes	1895	2109	1034	837	686
Hypertension	1265	1602	924	473	447
Thyroid disease	742	1248	625	436	296
Atopic diathesis	2076	2481	1219	1028	955
Anaemia	428	576	319	259	197
COPD	296	331	178	118	85
CKD	179	211	147	78	66
Liver disease	196	214	159	109	98

## Discussion

In this study, patients’ data were collected for five consecutive years, starting from 2018 to 2022, using the data extraction sheet. An increasing number of cases of dermatophytosis was noticed in this study, rising about 16% in 2019 compared to 2018, but a decline of 45% in cases was observed in 2020 compared to 2019. The rise in the cases of dermatophytosis in India was like an epidemic from 2014 onward, which is described in a study by Verma et al. [6]. In 2020, during the COVID-19 pandemic, various lockdown measures came into effect, congregation of people was not possible, and people staying at home mainly focused on their personal hygiene. Increased awareness of various skin infections was likely due to various types of social media, and people might have sought early treatment by visiting pharmacies, physicians, or tele-consultations. This could have contributed to the stiff decline in cases in 2020. Furthermore, prompt treatment by general physicians at the ground level healthcare system and making patients aware of avoidance of contributing factors could have played a significant role in the gradual decline of the cases over the years, showing a drop of 33% cases in 2021 compared to 2020 and 23% cases in 2022 compared to 2021.

This study showed that dermatophytosis was most commonly observed in the age group of 21-30 years (28.64%). Similar results were obtained by studies done by Verenkar et al. [7] and Sumana and Singaracharya [8]. This age group mostly belongs to the hostel students and working population, who could have contributed to the surge in the cases in this age group. People who have outdoor work, such as farmers and laborers, have more chances of contracting dermatophytosis from the environment. Similarly, students who stay in overcrowded and unhygienic hostel rooms also have a higher chance of contracting infection from fellow students. However, findings in the study of Bindu and Pavithran suggested a higher prevalence in the age group of 11-20 years [9]. 

Over the years from 2018 to 2022, among the cases presenting with dermatophytosis, males have always outnumbered females with a ratio of 1.5:1, which was comparable to studies by Amin and Shah [10] and Singh and Beena [11] but contrasting from the study by Belurkar et al. [12]. The increased prevalence of dermatophytosis in males may be due to the occupational work outdoors, which may lead to an increased risk of exposure to infections and hot-humid climatic conditions facilitating the growth of dermatophytes. Social stigma plays a pivotal role in females in the rural population leading to underreporting, which further leads to lower incidence. 

Patients were classified into three groups according to the duration of symptoms, i.e., less than one month, one to three months, and more than three months. In our study, 20.63% had a disease duration of less than one month, 26.29% had within one to three months, and 53.07% had a duration of more than three months. In an earlier study, Kumar et al. noticed the duration of symptoms to be less than one month in 13% of the patients, one to three months in 33.7% of patients, and more than three months in 53.3% of the patients [13]. A lengthened duration of illness of six months and beyond was obtained in 46.4% of the patients, which was similar to the studies by Mahajan et al. [14], where it was around 53.9%, and Agarwal et al. [15], where it was 62.5%. The possible reasons for such chronicity could be due to insufficient anti-fungal dosing, irregular treatment, and use of steroid-containing creams, which decrease inflammation and pruritus and help in fungal growth and proliferation. 

Both urban and rural population run similar risks of contracting dermatophytes. In our study, the rural predominance was observed due to the raised frequency of agricultural work leading to increased perspiration, which was also observed a study conducted by Hanumathappa et al. [16]. However, in the last three years of our study, the scenario shifted toward increasing the involvement of the urban population. The rationale behind this might be due to increased health-seeking behavior in the urban population seeking remedies at the earliest. However, the easy availability of an irrational combination of steroid, antibacterial, and antifungal creams in urban areas might be instrumental in urban predominance. 

People who work outdoors in hot and humid conditions are at significant risk of infection since it favors a suitable environment for the growth of dermatophytes. Many studies have reported that manual laborers are frequently affected [17,18]. Farmers also have increased risk due to exposure to fungus from the environment (soil and animals). While farmers and laborers were among the most affected in 2018 and 2019, students and homemakers were the most susceptible groups observed in 2020. Tight-fitting synthetic clothes, wearing footwear for a long time, and overcrowding in hostel rooms are possible contributing factors in students, which was mentioned in the studies conducted by Agarwal et al. [15] and Sharma et al. [19]. The increased sweating in the hot environment of the kitchen hastens the growth of dermatophytes, mostly over the body folds and waistline in Indian homemakers. Homemakers were the most commonly affected group (25.1%) in the study by Rudramurthy et al. [20]. 

In the current study, a family history of dermatophytosis was present in 27% of cases. A comparable result was also demonstrated in the studies by Mahajan et al. [14], where a positive family history was present in 30.9% of cases, and Vineetha et al. [17], where it was seen in 21%. Overcrowding and sharing of clothes, footwear, and combs were significant factors in the household transmission of dermatophytes. 

Tinea corporis was the most common clinical type observed, involving around 34.34% of cases, which is similar to the studies by Agarwal et al. [15], Vineetha et al. [17], and Noronha et al. [21], and features of both tinea cruris and tinea corporis were seen in 22.24% of cases. Tinea cruris variant was observed in 18.23% of cases. In this study, tinea unguium was seen in 4.7% of cases, which is analogous to the studies by Singh and Beena (1.9%) [11] and Karmakar et al. (2.8%) [22]. However, Bindu et al. reported about 13.3% of cases in their study [9]. This variation could be attributed to the predominant age group and other associated systemic co-morbidities like diabetes. 

In all, 5467 specimens were sent for KOH mount examination over five years, of which 3977 (72.75%) cases were positive for fungal elements. However, 431 specimens were sent for culture over five years, while 236 cases (54.77%) were culture-positive. Most common organisms isolated over 2018 and 2019 were *T. mentagrophytes* (61.98%), *T. rubrum* (22.92%), and *M. audouinii *(7.81%). From 2020 to 2022, common isolates are *T. mentagrophyte* (27.5%), *T. rubrum* (31.67%), and *T. tonsurans* (15%).

Out of the chronic and recurrent cases where various laboratory investigations were conducted, diabetes, thyroid disease, and chronic kidney disease were found to be the prevalent co-morbidities. Atopic diathesis was the major co-morbidity present in 9.94% of cases, comparable to the study by Bindu and Pavithran [9]. Low blood perfusion in anemia cases and profuse sweating in hyperthyroid diseases could have contributed to chronicity among tinea unguium and tinea cruris/corporis cases. Most of the erythrodermic variants were patients of COPD, CKD, and other chronic diseases who were on oral steroid therapy for a long duration. 

Limitations

This was a single-center record-based study. Multicentric studies with follow-up should be carried out to determine the actual burden and trends of the disease.

## Conclusions

The findings of this study showed an initial increase in trend from 2018 to 2019 (116%) and, thereafter, a downward one from 2019 to 2022. The most common age group affected was 21-30 years (28.64%), of which a majority (53.07%) of patients had a disease duration of over three months. There was an urban predominance of dermatophytosis from 2020. Tinea corporis was the commonest clinical variant (34.34%), while atopic diathesis was a major co-morbidity (9.94%). The rationality behind the downward trend from 2020 could have been factors like COVID-19-related factors, public awareness, a better understanding of the disease, and prompt management at various levels by healthcare providers. Similar types of studies may be carried out over different parts of our country to assess the actual burden of the disease and expert planning for the containment of the disease in a more competent manner.

## References

[1] Gupta CM, Tripathi K, Tiwari S, Rathore Y, Nema S, Dhanvijay AG (2014). Current trends of clinico mycological profile of dermatophytosis in Central India. IOSR J Dent Med Sci.

[2] Dogra S, Uprety S (2016). The menace of chronic and recurrent dermatophytosis in India: is the problem deeper than we perceive?. Indian Dermatol Online J.

[3] Sentamilselvi G, Kamalam A, Ajithadas K, Janaki C, Thambiah AS (1997). Scenario of chronic dermatophytosis: an Indian study. Mycopathologia.

[4] Panda S, Verma S (2017). The menace of dermatophytosis in India: the evidence that we need. Indian J Dermatol Venereol Leprol.

[5] Das K, Pingali MS, Paital B (2021). A detailed review of the outbreak of COVID-19. Front Biosci (Landmark Ed).

[6] Verma SB, Panda S, Nenoff P (2021). The unprecedented epidemic-like scenario of dermatophytosis in India: I. Epidemiology, risk factors and clinical features. Indian J Dermatol Venereol Leprol.

[7] Verenkar MP, Pinto MJ, Rodrigues S, Roque WP, Singh I (1991). Clinico‑microbiological study of dermatophytoses. Indian J Pathol Microbiol.

[8] Sumana V, Singaracharya MA (2004). Dermatophytosis in Khammam (Khammam district, Andhra Pradesh, India). Indian J Pathol Microbiol.

[9] Bindu V, Pavithran K (2002). Clinico‑mycological study of dermatophytosis in Calicut. Indian J Dermatol Venereol Leprol.

[10] Amin A G, Shah HS (1973). Dermatophytosis. Indian J Dermatol.

[11] Singh S, Beena PM (2003). Profile of dermatophyte infections in Baroda. Indian J Dermatol Venereol Leprol.

[12] Belurkar DD, Bharmal RN, Kartikeyan S, Vadhavkar RS (2004). A mycological study of Dermatophytoses in Thane. Bombay Hosp J.

[13] Kumar Y, Singh K, Kanodia S, Singh S, Yadav N (2015). Clinico‑epidemiological profile of superficial fungal infections in Rajasthan. Med Pulse‑Int Med J.

[14] Mahajan S, Tilak R, Kaushal SK, Mishra RN, Pandey SS (2017). Clinico-mycological study of dermatophytic infections and their sensitivity to antifungal drugs in a tertiary care center. Indian J Dermatol Venereol Leprol.

[15] Agarwal US, Saran J, Agarwal P (2014). Clinico-mycological study of dermatophytes in a tertiary care centre in Northwest India. Indian J Dermatol Venereol Leprol.

[16] Hanumanthappa H, Sarojini K, Shilpashree P, Muddapur SB (2012). Clinicomycological study of 150 cases of dermatophytosis in a tertiary care hospital in South India. Indian J Dermatol.

[17] Vineetha M, Sheeja S, Celine MI, Sadeep MS, Palackal S, Shanimole PE, Das SS (2018). Profile of dermatophytosis in a tertiary care center. Indian J Dermatol.

[18] Hazarika D, Jahan N, Sharma A (2019). Changing trend of superficial mycoses with increasing nondermatophyte mold infection: a clinicomycological study at a tertiary referral center in Assam. Indian J Dermatol.

[19] Sharma R, Adhikari L, Sharma RL (2017). Recurrent dermatophytosis: a rising problem in Sikkim, a Himalayan state of India. Indian J Pathol Microbiol.

[20] Rudramurthy SM, Shankarnarayan SA, Dogra S (2018). Mutation in the squalene epoxidase gene of Trichophyton interdigitale and Trichophyton rubrum associated with allylamine resistance. Antimicrob Agents Chemother.

[21] Noronha TM, Tophakhane RS, Nadiger S (2016). Clinico-microbiological study of dermatophytosis in a tertiary-care hospital in North Karnataka. Indian Dermatol Online J.

[22] Karmakar S, Kalla G, Joshi KR, Karmakar S (1995). Dermatophytoses in a desert district of Western Rajasthan. Indian J Dermatol Venereol Leprol.

